# The effect of forensic events on health status and housing stability among homeless and vulnerably housed individuals: A cohort study

**DOI:** 10.1371/journal.pone.0211704

**Published:** 2019-02-07

**Authors:** Charles Walsh, Anita M. Hubley, Matthew J. To, Monica Norena, Anne Gadermann, Susan Farrell, Stephen W. Hwang, Anita Palepu

**Affiliations:** 1 Division of General Internal Medicine, Department of Medicine, University of British Columbia, Vancouver, BC, Canada; 2 Department of Education Counselling Psychology and Special Education, University of British Columbia, Vancouver, BC, Canada; 3 Centre for Urban Health Solutions, St. Michael’s Hospital, Toronto, ON, Canada; 4 Centre for Health Evaluation and Outcome Sciences, St. Paul’s Hospital, Vancouver, BC, Canada; 5 School of Population and Public Health, University of British Columbia, Vancouver, BC, Canada; 6 Royal Ottawa Health Care Group, Ottawa, ON, Canada; University of Indianapolis, UNITED STATES

## Abstract

We sought to characterize the association between a forensic event (arrest or incarceration) with housing vulnerability and mental and physical health status over a four-year follow-up among a cohort of homeless and vulnerably housed individuals in Vancouver, Toronto and Ottawa. Data were obtained from the Health and Housing in Transition Study, a prospective cohort study of homeless and vulnerably housed individuals between 2009 and 2012. Participants were interviewed in-person at baseline (N = 1190) and at four annual follow-up time points. We used generalized estimating equations to characterize the independent associations between a forensic event and the number of residential moves and SF-12 physical and mental health component scores over the four-year follow-up period. We analyzed data from 1173 homeless and vulnerably housed participants. Forensic events were reported by 446 participants at baseline. In multivariate analyses, a history of forensic event in the preceding twelve months was independently associated with an increased number of residential moves over the four-year follow-up period (ARR 1.24; 95% CI 1.19–1.3). It was not, however, independently associated with a change in physical or mental health status (respective ß-estimates; 95% CI: -0.34; -1.02, 0.34, and -0.69; -1.5, 0.2). Female gender and a history of problematic substance use were significantly associated with all three primary outcomes. This suggests arrest or incarceration is associated with increased housing vulnerability. The results underline the importance of supporting individuals experiencing arrest or incarceration with post-release planning in order to obtain stable housing after discharge.

## Introduction

The incarcerated population in Canada bears a disproportionate burden of physical and mental health problems compared to the general population [1]. Persons who experience incarceration are more likely to use both illicit and prescription drugs of abuse and have higher rates of infectious diseases, cardiovascular disease, and cancers [1,2]. Their rates of psychosis, depression, and personality disorders are also higher [1,2]. Homelessness increases risk of incarceration with the rates of homelessness prior to incarceration as much as five times higher than the general population [3,4]. One study based on a US national survey of incarcerated persons found that 15% had been homeless in the year prior to incarceration, or eleven times the estimate for the general US population [5]. The same study found that incarcerated persons who have a history of homelessness were more likely to have been cited for both violent and non-violent offenses, to have higher rates of mental health and substance use issues, and to be unemployed [5]. It is clear not only that homelessness is a risk factor for incarceration, but also that incarceration is a risk factor for homelessness [5]. The transition back into the community after discharge therefore represents an aspect of public health that deserves attention. A successful reintegration can improve health, reduce crime, and decrease costs to society [6]. Though health may initially improve upon incarceration, research suggests that health often declines significantly after discharge [6,7]. With over 30 million incarcerated persons being released each year worldwide, greater attention to this critical transition point could mean the difference between successful reintegration or further crime and poor health [7].

In order for an individual to be successful after discharge from a correctional facility, housing is one of the most basic needs that must be fulfilled [8]. Indeed, given research that suggests homelessness is a risk factor for incarceration, ensuring housing following discharge could reduce recidivism [3]. Unfortunately, current research suggests that formerly incarcerated persons are up to six times more likely to be homeless following release than the general population [9]. The Urban Institute's *Returning Home* project found that over half of all incarcerated persons discharged in Ohio moved two or more times during the first year [10]. In the Canadian context, approximately 30% of incarcerated persons are discharged without secure housing [4,11]. However, these statistics fail to capture the housing vulnerability that these individuals may experience over a longer term. There remains a significant gap in our understanding of longer-term housing vulnerability, with few prospective studies following discharged individuals for over two years [1,6,12].

Individuals recently released from corrections experience sub-optimal health outcomes, including poorly controlled chronic diseases, drug overdose, preventable hospitalization, and mortality [6]. Some research suggests their mortality rate may be as much as 30 times higher than the general population [1]. Furthermore, incarcerated individuals with a history of mental health problems are four times more likely to die of suicide or drug overdose than those without a mental health history in the six months following release [12]. A more comprehensive understanding of the impacts of incarceration on physical and mental health is still lacking, especially in the Canadian context [1,4,6]. Research to clarify the morbidity and mortality of incarcerated individuals upon release is scant. Studies thus far are largely retrospective or focus on outcomes during incarceration, rather than following individuals after release [1,4]. Furthermore, there are no published prospective studies on the effects of arrest without incarceration on health and housing status. This study therefore sought to add more broadly to our understanding of the impact of arrest and incarceration on housing vulnerability as well as physical and mental health status.

Using prospective cohort data collected in Vancouver, Toronto, and Ottawa, Canada, we aimed to characterize the association between a forensic event (incarceration or arrest) and (a) physical and mental health status, and (b) number of residential moves after discharge (as a proxy for housing vulnerability).

## Methods

The findings that we present here are derived from data collected by the Health and Housing in Transition (HHiT) study. This is a prospective cohort study that investigated the health and housing status of homeless and vulnerably housed persons in Vancouver, Toronto, and Ottawa beginning in 2009, with four annual follow-up interviews. Further methodological details have been published previously [13]. The sample size of this study differs from the methodological description [13] because two participants were subsequently noted to have participated in the study twice, and their duplicates were therefore removed.

### Participants and recruitment

The 2009 HHiT study recruited 1190 homeless and vulnerably housed single adults (aged 18 years or older). Homelessness was defined as not having lived in one’s house in the previous week but instead having lived in a shelter, public space, vehicle, abandoned building, or someone else’s home. Vulnerably housed was defined as having lived in one’s own home but having been homeless and/or having had two or more moves in the preceding twelve months. Homeless participants were recruited at shelters and meal programs, making use of sampling methods designed for this population [14]. Rooming houses, single room occupancy hotels, meal programs, drop-in centres, and community health centres were all used to recruit vulnerably housed participants. At baseline, 595 participants reported being homeless and 595 reported being vulnerably housed. All participants provided written informed consent and were compensated $20 for completing each annual interview. This study was approved by the Research Ethics Boards at St. Michael’s Hospital in Toronto, University of Ottawa, and the University of British Columbia. This study used data from the baseline survey and four follow-up interviews.

### Survey instrument

Trained interviewers administered the 60–90 minute survey. Sociodemographic data, including age, gender, and self-reported ethnicity were collected, as were employment, arrests, and incarceration histories over the preceding twelve months. For participants’ housing history, we classified data based on methods adapted from Tsemberis et al. [15]. Each residence in a participant’s housing history was classified into one of 25 types of residence, which were then categorized into one of three mutually exclusive residence categories: housed, institution, and homeless. Institutions referred to correctional facilities, hospitals, residential substance use treatment, halfway housing, or long-term/nursing home. Periods of time spent in institutions were further classified into residential states of functionally housed or functionally homeless based on their residence before and after the institutional stay using a functional classification as previously described [16]. For example, a participant would be functionally homeless if they reported the following type of residences: staying with friends/family but not paying rent; homeless shelter; homeless on streets; living in a campground. Participants were also categorized as functionally homeless if they were categorized as being in an institution and prior to that, they were homeless or after being in the institution, they were homeless. The number of residential moves in the preceding year was derived based on the housing history for each participant.

Participants were also asked in the baseline interview to identify the cumulative number of lifetime chronic health conditions (0, 1, 2, ≥3) diagnosed by a health care practitioner that had lasted at least 6 months, if they had ever been diagnosed with a mental health problem, and their lifetime duration of being homeless. Problematic substance use was assessed at each interview. The ten-item Drug Abuse Screening Test (DAST-10) was used to screen for drug use, with a DAST-10 score of ≥6 classified as problematic drug use [17]. The Alcohol Use Disorders Identification Test (AUDIT), a ten-item questionnaire, was used to screen for alcohol use disorder, where a score of ≥20 was classified as problematic alcohol use [18]. These instruments have been validated previously for their assessment of vulnerable populations [19,20].

### Main explanatory variable

A forensic event was defined as having responded yes to either: “In the past 12 months, were you arrested by the police?” or “In the past 12 months were you incarcerated, whether in preventive detention, prison or a penitentiary?” These data were collected at each of the five interviews.

### Outcome variables

We used the number of residential moves the participant experienced during the year prior to the survey interview to assess housing stability. We defined number of residential moves as captured from the housing history in the preceding year. We derived the physical component score (PCS12) and mental component score (MCS12) from the Short Form 12-item health survey (SF-12) [21], as measures of physical and mental health status, respectively. The SF-12 has been widely used and shown to have adequate reliability and validity in diverse samples including individuals with severe mental illness [22,23] and homeless individuals [24].

### Statistical analysis

We compared groups using the chi-square test for categorical variables and one-way ANOVA or Kruskall Wallis nonparametric test for continuous variables. We used the bivariate and multivariate generalized estimating equation (GEE) to analyze the association between forensic events during the preceding twelve months and three primary outcomes: physical health status as measured by the physical component score (PCS12), mental health status as measured by the mental component score (MCS12), and housing vulnerability as measured by the number of residential moves over the year preceding the interview.

GEE models were used to account for serial observations of the same participants. We adjusted for the correlation between measures over time using an exchangeable correlation structure [25,26]. PCS12 and MCS12 were both modeled as continuous outcomes using multiple linear regression and the number of residential moves was modeled as a count using a Poisson regression model. Age, sex, city, and ethnicity (categorized as White vs. others due to small sample size) were demographic covariates forced in the final adjusted models. For the multivariate analyses, we excluded lifetime homeless duration because it did not have an independent association with any of the primary outcomes.

Each multivariate model was adjusted for age, sex, problematic substance use, employment, city, ethnicity (White vs. others), being homeless at interview point and a categorical interview time point variable, as responses were obtained at each follow-up interview time point (years 1, 2, 3 and 4). When modeling mental health status, we adjusted for having 3 or more chronic health conditions at baseline and when modeling physical health status, we adjusted for lifetime history of a mental health problem at baseline. When modeling the number of residential moves, we adjusted for both having 3 or more chronic health conditions and lifetime history of a mental health problem at baseline as neither variable was highly correlated with the outcome variable definition. In a supplementary analysis, we also fit models examining the effect of arrest and incarceration independently on number of residential moves. In addition, we performed a lagged analysis that related forensic events in a given year to number of residential moves in the subsequent year.

## Results

**Fig 1:**
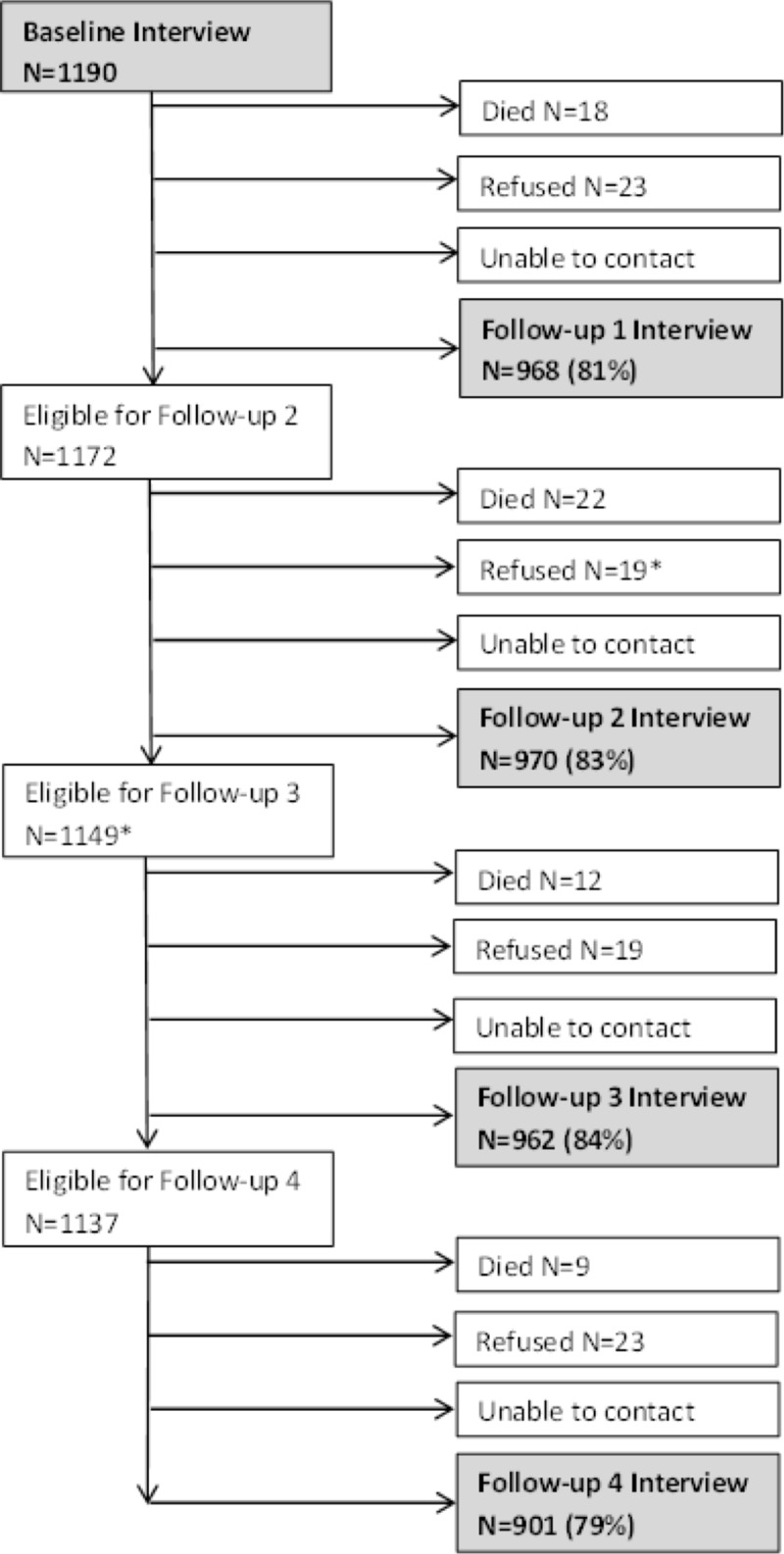
Health and Housing in Transition Study Participant Status at each Follow-up Interview. Vancouver, Toronto, and Ottawa 2009–2013. * 1 participant withdrew from the study during Follow-up 2.

The participant flow over the 4 years of follow-up from baseline is depicted in Fig 1 where we achieved an overall follow-up rate of 79%. In this analysis, we included 1173 participants (17 individuals identifying as transgender were excluded due to small numbers). There were 446 participants (38%) who reported a history of a forensic event in the preceding twelve months at baseline and their characteristics are reported in Table 1. We found that, at baseline, participants with a forensic event history were younger, had slightly lower PCS and MCS, had a higher number of residential moves in the previous year, were almost twice as likely to report problematic substance use, were more likely to report having been diagnosed with a mental health problem, and were more likely to have 3 or more chronic health conditions compared to those without a forensic history. The scores on the SF-12 scales are expressed as T-scores (mean of 50 and standard deviation of 10, range 0–100). Thus, the means of physical and mental health status of both groups were below average compared to U.S. population norms. Of note, the proportion of participants reporting forensic events declined over the four-year follow-up period from 38% at baseline to 21% at year 4; the magnitude of the decline was similar for arrests and incarceration (Table 2).

**Table 1:** Baseline participant characteristics by forensic event.

	Forensic Event		
	Yes (n = 446)	No (n = 723)	P-value
Physical Component Score (mean, SD)	43.8 (10.9)	44.9 (11.5)	0.01
Mental Component Score (mean, SD)	38.1 (12.3)	39.8 (13.4)	0.03
Number of residential moves (mean, SD)	3.0 (1.3)	2.7 (1.1)	**<0.01**
Age (mean, SD)	39.6 (9.6)	43.9 (10.8)	**<0.01**
Female (N, %)	135 (30.3)	250 (34.6)	0.13
Ethnicity			0.57
White (N, %)	278 (63.9)	437 (62.3)	
Others (N, %)	157 (36.1)	265 (37.7)	
City			0.02
Toronto (N, %)	129 (28.9)	261 (36.1)	
Ottawa (N, %)	169 (37.9)	225 (31.1)	
Vancouver (N, %)	148 (33.2)	237 (32.8)	
Employed in past 12 months (N, %)	168 (37.7)	298 (41.3)	0.22
Lifetime homeless duration (mean, SD)	6.0 (6.3)	4.8 (5.9)	**<0.01**
Residential State at interview time point			0.38
Vulnerably Housed (N, %)	213 (47.9)	365 (50.5)	
Homeless (N, %)	232 (52.1)	358 (49.5)	
Problematic Substance use (N, %)	238 (53.4)	198 (27.5)	**<0.01**
Ever had a mental health problem (N, %)	250 (57.1)	341 (47.7)	**<0.01**
3 or more chronic health conditions (N, %)	251 (56.3)	330 (45.6)	**<0.01**

p-values <0.01 are **bolded**

**Table 2:** Number of forensic events by interview time point.

Interview Period	Arrested N (%)	Incarcerated N (%)	Forensic Event N (%)
Baseline	421 (36)	333 (29)	446 (38)
Year 1	274 (29)	212 (22)	294 (31)
Year 2	226 (24)	184 (19)	248 (26)
Year 3	193 (20)	158 (17)	208 (22)
Year 4	176 (20)	137 (16)	187 (21)

Table 3 presents the bivariate and multivariate GEE linear regression model findings for the factors associated with physical health status as measured by the PCS12. Results of the multivariate model indicate that factors significantly associated with worse physical health status were older age (ß = -2.46; 95% CI: -2.95, -1.97), female sex (ß = -2.58; 95% CI: -3.7, -1.46), problematic substance use (ß = -1.07; 95% CI:-1.77, -0.36), a history of mental health problems (ß = -2.36; 95% CI:-3.4, -1.33), and living in Vancouver relative to Toronto (ß = -1.61; 95% CI:-2.79, -0.42). Being employed was significantly associated with better physical health status (ß = 2.15; 95% CI: 1.47, 2.83). Having a forensic event was not independently associated with physical health status (ß = -0.34; 95% CI: -1.02, 0.34).

**Table 3:** Multivariate GEE linear regression model to estimate the independent effect of having a forensic event on physical health status (PCS12).

Independent Variables		PCS12 at baseline Mean (SD)	Unadjusted ß-Estimates for PCS12 baseline to FU4 interview (95% CI)	Adjusted ß-Estimates for PCS12 baseline to FU4 interview (95% CI)
Forensic event*	Yes	43.8 (10.9)	0.19 (-0.47, 0.84)	-0.34 (-1.02, 0.34)
	No	44.9 (11.5)		
				
Age (by decade)			**-2.40 (-2.9, -1.9)**	**-2.46 (-2.95, -1.97)**
Sex	Female	42.0 (11.4)	**-3.02 (-4.16, -1.88)**	**-2.58 (-3.70, -1.46)**
	Male	45.7 (11.1)	** **	** **
Employment*	Yes	47.5 (10.8)	**2.57 (1.92, 3.22)**	**2.15 (1.47, 2.83)**
	No	42.6 (11.2)	** **	** **
City:			** **	** **
	Ottawa	44.7 (11.7)	-0.51 (-1.84, 0.82)	-0.78 (-2.08, 0.51)
Vancouver		43.7 (11.5)	-1.30 (-2.56, -0.05)	**-1.61 (-2.79, -0.42)**
	Toronto	45.1 (10.7)	Reference	Reference
Ethnicity	White	44.5 (11.3)	-0.06 (-1.17, 1.05)	0.18 (-0.89, 1.24)
	Other	44.6 (11.2)		
Homeless at interview time point*	Yes	45.4 (11.7)	**0.94 (0.25, 1.63)**	0.69 (-0.01, 1.39)
	No	43.5 (10.8)	** **	
Interview time point*			**-0.26 (-0.44, -0.08)**	-0.14 (-0.33, 0.04)
Lifetime homeless duration			-0.05 (-0.13, 0.04)	
Problematic substance use*	Yes	44.1 (10.9)	-0.82 (-1.5, -0.13)	**-1.07 (-1.77, -0.36)**
	No	44.7 (11.6)		** **
Ever had a mental health diagnosis	Yes	42.5 (11.2)	**-2.93 (-4, -1.87)**	**-2.36 (-3.40, -1.33)**
	No	46.7 (11.1)		** **
3 or more chronic conditions	Yes	39.2 (10.4)	**-9.18 (-10.11, -8.24)**	** **
	No	49.8 (9.5)	** **	** **

*Time varying covariates

Multivariate regression missing data (248/4919 = 5%)

Coefficients with p-values < 0.05 are bolded

The multivariate GEE linear regression model findings for factors associated with mental health status as measured by the MCS12 are presented in Table 4. Factors significantly associated with worse mental health scores included female sex (ß = -2.3; 95% CI: -3.48, -1.12), problematic substance use (ß = -4.63; 95% CI: -5.46, -3.8), having 3 or more chronic health conditions (ß = -2.79; 95% CI: -3.91, -1.67), and being functionally homeless at the interview time point (ß = -2.7; 95% CI: -3.56, -1.85). On average, the MCS12 improved over time (ß = 0.71; 95% CI: 0.48, 0.94). A history of a forensic event was not independently associated with mental health status (ß = -0.69; 95% CI: -1.5, 0.12).

**Table 4:** Multivariate GEE linear regression model to estimate the independent effect of having a forensic event on mental health status (MCS12).

Independent Variables		MCS12 at baseline Mean (SD)	Unadjusted ß-Estimates for MCS12 baseline to FU4 interview (95% CI)	Adjusted ß-Estimates for MCS12 baseline to FU4 interview (95% CI)
Forensic event*	Yes	38.1 (12.3)	**-2.42 (-3.20, -1.63)**	-0.69 (-1.50, 0.12)
	No	39.8 (13.4)	** **	
Age (by decade)			**0.71 (0.15, 1.26)**	0.52 (-0.02, 1.06)
Sex	Female	37.5 (13)	**-2.53 (-3.70, -1.36)**	**-2.30 (-3.48, -1.12)**
	Male	39.9 (13)	** **	** **
Employment*	Yes	39.6 (13.1)	0.76 (-0.01, 1.53)	0.73 (-0.05, 1.51)
	No	38.8 (13.0)		
City:			** **	
	Ottawa	38.3 (13.6)	-0.70 (-2.07, 0.66)	0.24 (-1.08, 1.56)
	Vancouver	38.7 (12.8)	-0.49 (-1.82, 0.83)	0.45 (-0.85, 1.74)
	Toronto	40.5 (12.5)	Reference	Reference
Ethnicity	White	39.3 (13.1)	-0.26 (-1.41, 0.9)	-0.85 (-1.97, 0.27)
	Other	38.9 (12.8)		
Homeless at interview time point*	Yes	38.5 (13.1)	**-3.57 (-4.40, -2.74)**	**-2.70 (-3.56, -1.85)**
	No	39.8 (13)	** **	** **
Interview time point*			**1.02 (0.80, 1.24)**	**0.71 (0.48, 0.94)**
Lifetime homeless duration			-0.04 (-0.14, 0.05)	
Problematic substance use*	Yes	34.8 (11.5)	**-5.41 (-6.23, -4.60)**	**-4.63 (-5.46, -3.80)**
	No	41.8 (13.2)	** **	** **
Ever had a mental health diagnosis	Yes	36.0 (12.4)	**-5.41 (-6.48, -4.34)**	
	No	42.4 (12.8)	** **	
3 or more chronic conditions	Yes	36.8 (12.8)	**-3.05 (-4.15, -1.95)**	**-2.79 (-3.91, -1.67)**
	No	41.5 (12.8)	** **	** **

*Time varying covariates

Multivariate regression missing data (248/4919 = 5%)

Coefficients with p-values < 0.05 are bolded

Table 5 presents the bivariate and multivariate GEE Poisson regression model results for factors associated with the number of residential moves. Factors independently associated with a higher number of residential moves over the follow-up period male sex (ARR 0.93; 95% CI: 0.89, 0.98), problematic substance use (ARR 1.17; 95% CI: 1.12, 1.22), living in Vancouver (vs. Toronto) (ARR 1.08; 95% CI: 1.03, 1.14), and being functionally homeless at the interview time point (ARR 1.25; 95% CI: 1.2, 1.31). Older age was associated with fewer residential moves (Adjusted rate ratio [ARR] 0.94; 95% CI: 0.92, 0.97), On average, number of residential moves declined over the follow-up period (ARR 0.83; 95% CI: 0.82, 0.85). A history of a forensic event in the preceding twelve months was independently associated with a higher number of residential moves (ARR 1.24, 95% CI 1.19–1.3). The results of examining the independent effects of arrest and incarceration on residential moves as well as the lagged analysis are presented in Tables A-C in S1 File. The main effects were essentially unchanged.

**Table 5:** Multivariate GEE Poisson regression model to estimate the independent effect of having a forensic event on the number of residential moves.

Independent Variables		Number of residential moves at baseline Median (IQR)	Unadjusted RR for number of residential moves baseline to FU4 interview (95% CI)	Adjusted RR for number of residential moves baseline to FU4 interview (95% CI)
Forensic event*	Yes	3 (2, 4)	**1.51 (1.44, 1.58)**	**1.24 (1.19, 1.3)**
	No	3 (2, 3)	** **	** **
Age (by decade)			**0.91 (0.88, 0.93)**	**0.94 (0.92, 0.97)**
Sex	Female	3 (2, 3)	**0.89 (0.84, 0.93)**	**0.93 (0.89, 0.98)**
	Male	3 (2, 3)	** **	** **
Employment*	Yes	3 (2, 4)	**1.14 (1.09, 1.20)**	1.05 (1.00, 1.10)
	No	3 (2, 3)	** **	
City:			** **	
	Ottawa	3 (2, 4)	**1.12 (1.05, 1.19)**	1.06 (1.01, 1.12)
	Vancouver	3 (2, 3)	**1.10 (1.04, 1.16)**	**1.08 (1.03, 1.14)**
	Toronto	2 (2, 3)	Reference	Reference
Ethnicity	White	3 (2, 4)	1.05 (1.00, 1.11)	1.05 (1.00, 1.10)
	Other	3 (2, 3)		
Homeless at interview time point*	Yes	3 (2, 3)	**1.53 (1.46, 1.60)**	**1.25 (1.20, 1.31)**
	No	3 (2, 4)		
Interview time point*			**0.81 (0.79, 0.82)**	**0.83 (0.82, 0.85)**
Lifetime homeless duration			1.00 (0.99, 1.00)	
Problematic Substance use*	Yes	3 (2, 4)	**1.33 (1.27, 1.4)**	**1.17 (1.12, 1.22)**
	No	3 (2, 3)	** **	** **
Ever had a mental health diagnosis	Yes	3 (2, 4)	1.02 (0.97, 1.07)	1.01 (0.97, 1.06)
	No	3 (2, 3)		
3 or more chronic conditions	Yes	3 (2, 4)	0.94 (0.90, 0.99)	0.97 (0.93, 1.02)
	No	3 (2, 3)		

*Time varying covariates

Multivariate regression missing data (236/4919 = 5%)

Coefficients with p-values < 0.05 are bolded

## Discussion

We found that participants with a history of forensic event (i.e., arrest or incarceration) had significantly more residential moves during the four-year follow-up period. This held true when adjusting for covariates including age, ethnicity, employment, problematic substance use, chronic health conditions, and a lifetime history of mental health diagnosis. Our findings further support previous research showing that incarceration destabilizes housing over the long term [9,10,11,27]. To et al. examined the effect of incarceration on housing stability and found that homeless and vulnerably housed participants were less likely to be housed over a two-year follow-up period [28]. Our study further supports this finding by looking at four years of follow-up data, and by using more granular measures of housing (residential moves), rather than a simple binary of housed versus homeless status at each interview time point [28].

We did not find that a forensic event showed significant associations with altered mental and physical health status over time, as measured by the MCS12 and PCS12, after adjustment for confounders. Of note, our results indicate that participants with and without a history of forensic events rated their physical and mental health status as lower than the general population. Participants in both groups scored about half a standard deviation lower than U.S. population norms on the PCS12 and about one standard deviation lower on the MCS12. Furthermore, participants with a history of forensic event at baseline also had lower MCS12 and PCS12 scores compared to those without a history of forensic event. Previous studies have demonstrated that incarceration is associated with increased substance use rates and worsened mental health outcomes [1–4]. Our study's inability to demonstrate an association between incarceration or arrest and altered mental or physical health functioning over time may be related to the difference in baseline health. Our study used different measures of physical and mental health than previous studies and included arrest in addition to incarceration as the main explanatory variable; comparability may therefore be limited [1–4]. Large, prospective studies that follow incarcerated individuals after release are needed to investigate for a link between incarceration and physical and mental health outcomes over time [3]. Our findings of higher baseline rates of reported mental health diagnoses, chronic physical health conditions, and substance use among homeless individuals and individuals with a history of incarceration, as compared to the general population, are consistent with research to date [1–4].

Incarceration may result in loss of housing or make it more difficult to find housing after prison release, whether as a result of poor discharge planning or reluctance of landlords to accept tenants with a criminal record [28]. More effort is needed to secure stable housing for incarcerated persons prior to discharge. By discharging individuals directly to supportive housing, New York City’s Housing First policy has reduced recidivism, in addition to reducing emergency department usage rates and hospital admissions [29]. In the Canadian context, the At Home/Chez Soi study on a population of homeless adults with mental illness in five Canadian cities compared a Housing First approach with treatment as usual (generally mental health or addiction treatment first, then housing) [30]. Study authors were able to demonstrate an approximate 40% increase in average time stably housed among participants in the Housing First group [30]. Given that our study demonstrates that housing can remain vulnerable years after incarceration or arrest, any community supports put in place to help formerly incarcerated persons find or retain stable housing should remain available over the long term.

Participants from Vancouver were more likely to have more residential moves over the follow up period than participants from Toronto. This could relate to differences in housing availability or support services between the two cities. For example, the 2010 Vancouver Olympic Games saw developers develop profitable accommodations for visitors, thereby gentrifying the neighborhoods where affordable housing was located [13]. This resulted in a loss of low-cost housing options during our follow up period [13]. Other reasons, such as differences among participants in substance use or mental health, or differing availability of housing or support services across the cities, may be at play.

It is noteworthy that female sex and a history of problematic substance use were both significantly associated with each of our three outcomes in the three multivariate analyses. Review of the literature around the status of health for homeless women suggests that they experience higher rates of a myriad of health conditions, including malnutrition, infections, and abuse, compared to women in general [31]. One Toronto study demonstrated a mortality rate 30 times higher than the general population; the same study showed young homeless women appear to have poorer health status even compared to young homeless men [32]. However, this trend appeared to reverse for older age groups. Strong evidence also suggests that homeless women experience poorer mental health status than homeless men [33]. Because the majority of the homeless population in Canada is male, services available to homeless people are largely male-centric [34]. These services may not, therefore, adequately address the specific needs of homeless women, such as supports for victims of violence. This may, in part, explain the health disparity we observed in this study between men and women. Finally, there appears to be a paucity of research investigating the housing status of women following incarceration or arrest.

Research on problematic substance use has consistently demonstrated significant negative associations with mental and physical health status and housing stability [35,36]. Increased rates of hepatitis, HIV, and sepsis account for some of the physical health effects, while rates of depression and psychosis are also clearly higher [34]. Being stably housed appears to be associated with reduced substance use rates, with one retrospective analysis demonstrating significantly lower rates of substance use among formerly incarcerated individuals living in regulated housing compared to those in more vulnerable housing [37]. Another retrospective analysis in Quebec reviewed outcomes among a population of persons with mental illness who were incarcerated and discharged to supportive housing as opposed to independent housing. The study found that the persons discharged to independent housing were far more likely to commit a new offense and even to be readmitted for psychiatric treatment [38]. This is relevant to our study population given the prevalence of mental health diagnoses in our cohort. Evidence also suggests problematic substance use impedes access to stable housing [36]. Housing First programs have also been successful in improving housing stability among persons with substance use disorders and mental illness [30,39,40].

Lifetime homeless duration was not significantly associated with any of the primary outcomes. While surprising, we posit that the effects of longer duration of homelessness may be mediated via the significant association of problematic substance use, mental health issues, and chronic health conditions with the primary outcomes.

## Limitations and strengths

The study has several limitations, which need to be considered when interpreting the findings. Data from participants were self-reported and thus may be subject to recall bias and other forms of reporting bias. A response rate of 79% means 21% of study participants did not participate in the fifth interview. It is possible that non-participants were disproportionately destabilized in terms of housing or health, or both, and were therefore less likely to follow up. Furthermore, the study sample was limited to single adults and, therefore, is not entirely representative of the homeless populations of Vancouver, Toronto and Ottawa. Our data around arrest did not capture granular details such as length of time in custody, although we found that most arrests did result in incarceration. Such differences could presumably impact housing stability and would be useful to include in future studies. There were more total forensic events reflected in the composite outcome than accounted for by arrests alone. This indicates that approximately 10–20 participants each year reported incarceration without arrest if they were incarcerated from an arrest more than one year prior at the time of the interview (e.g. arrested in year 1 and incarcerated in year 2). Finally, while every attempt was made to adjust for confounders, we may not have accounted for all factors that influence the association between a forensic event and physical and mental health scores and the number of residential moves.

Strengths of this study include the retention methods used [41], large sample size from three cities across Canada, and a high follow-up rate of 79% over four years. Furthermore, this is one of the only large, prospective studies to follow persons who are homeless or vulnerably housed with reported incarceration in the previous year for an extended follow-up period.

## Conclusion

In summary, in this longitudinal study of homeless or vulnerably housed participants, we demonstrated that persons with a recent history of forensic events experience a higher number of residential moves over a four-year follow-up period. This suggests that incarceration or arrest can lead to longer term housing vulnerability. We did not, however, demonstrate a significant relationship between a recent history of forensic events (i.e., arrest, incarceration) and participants’ mental and physical health status. The results highlight the importance of providing support to individuals experiencing arrest or incarceration in obtaining stable housing not only in the short-run after discharge and post-release planning, but in the long-term as well.

## Supporting information

S1 File (DOCX)

S1 Dataset (XLSX)

## References

[1] Fazel S, Baillargeon J. The health of prisoners. The Lancet 2011; 377: 956–65. 10.1016/S0140-6736(10)61053-7 21093904

[2] McNiel DE, Binder RL, Robinson JC. Incarceration associated with homelessness, mental disorder, and co-occurring substance abuse. Psychiatr Serv 2005; 56(7): 840–6. 10.1176/appi.ps.56.7.840 16020817

[3] Tsai J, Rosenheck RA, Kasprow WJ, McGuire JF. Homelessness in a national sample of incarcerated veterans in state and federal prisons. Adm Policy Ment Health 2014; 41(3): 360–7. 10.1007/s10488-013-0483-7 23512110

[4] Kouyoumdjian F, Schüler A., Hwang S, Matheson F. Research on the health of people who experience detention or incarceration in Canada: a scoping review. BMC Public Health 2015;15: 419 10.1186/s12889-015-1758-6 25943182 PMC4443600

[5] Greenberg G, Rosenheck R. Jail Incarceration, Homelessness, and Mental Health: A National Study. Psychiatr Serv 2008; 59 (2): 170–7. 10.1176/ps.2008.59.2.170 18245159

[6] Kinner SA, Wang EA. The case for improving the health of ex-prisoners. Am J Public Health 2014; 104(8): 1352–1355. 10.2105/AJPH.2014.301883 24922122 PMC4103236

[7] Alves J. Maia Â, Teixeira F. Health conditions prior to imprisonment and the impact of prison on health: Views of detained women. Qual Health Res 2016; 26(6): 782–792. 26631680 10.1177/1049732315617217

[8] Hwang SW. Homelessness and health. CMAJ 2001;164(2):229–33. 11332321 PMC80688

[9] Greenberg GA, Rosenheck RA. Homelessness in the state and federal prison population. Criminal Behaviour and Mental Health 2008; 18: 88–103. 10.1002/cbm.685 18383201

[10] Visher CA, Courtney S. One year out: Experiences of prisoners returning to Cleveland Washington, DC: Urban Institute Justice Policy Center, 2007 Available at: http://www.urban.org/research/publication/one-year-out-experiences-prisoners-returning-cleveland (Accessed May 21, 2017).

[11] Canada Mortgage and Housing Corporation. Housing Options upon Discharge from Correctional Facilities. Ottawa, ON: CMHC, 2007. Available at: https://www03.cmhc-schl.gc.ca/catalog/productDetail.cfm?cat=126&itm=1&lang=en&fr=1450976072236 (Accessed May 22, 2017).

[12] Cutcher Z, Degenhardt L, Alati R. Poor health and social outcomes for ex‐prisoners with a history of mental disorder: a longitudinal study. Aust NZ J Public Health 2014; 38: 424–429. 10.1111/1753-6405.12207 24962322

[13] Hwang SW, Aubry T, Palepu A. The health and housing in transition study: A longitudinal study of the health of homeless and vulnerably housed adults in three Canadian cities. Int J Public Health 2011; 56(6): 609–623. 10.1007/s00038-011-0283-3 21858461

[14] Ardilly P, Le Blanc D. Sampling and weighting a survey of homeless persons: a French example. Survey Methodology 2001; 27(1): 109–18.

[15] Tsemberis S, McHugo G, Williams V, Hanrahan P, Stefancic A. Measuring homelessness and residential stability: The residential time-line follow-back inventory. J Community Psychol 2007; 35(1): 29–42

[16] The Health and Housing in Transition (HHiT) Study Team. Housing definitions and housing variables. University of Toronto TSpace https://tspace.library.utoronto.ca/handle/1807/69938. Published 2015.

[17] Yudko E, Lozhkina O, Fouts A. A comprehensive review of the psychometric properties of the drug abuse screening test. J Subst Abuse Treat 2007; 32(2): 189–198. 10.1016/j.jsat.2006.08.002 17306727

[18] Maisto SA, Carey MP, Carey KB, Gordon CM, Gleason JR. Use of the AUDIT and the DAST-10 to identify alcohol and drug use disorders among adults with a severe and persistent mental illness. Psychol Assess 2000; 12(2): 186–192. 10887764 10.1037//1040-3590.12.2.186

[19] Babor TF, Higgins-Biddle JC, Saunders JB, Monteiro MG. AUDIT: The alcohol use disorders identification test: Guidelines for use in primary care World Health Organization 2001.

[20] Donovan DM, Kivlahan DR, Doyle SR, Longabaugh R, Greenfield SF. Concurrent validity of the alcohol use disorders identification test (AUDIT) and AUDIT zones in defining levels of severity among out-patients with alcohol dependence in the COMBINE study. Addiction. 2006; 101(12): 1696–1704. 10.1111/j.1360-0443.2006.01606.x 17156168

[21] Ware JEJ, Kosinski M, Keller SD. A 12-item short-form health survey: Construction of scales and preliminary tests of reliability and validity. Medical Care 1996; 34: 220–233. 8628042 10.1097/00005650-199603000-00003

[22] Luo X, Lynn George M, Kakouras I, Edwards CL., Pietrobon R, Richardson W, et al Reliability, validity and responsiveness of the short form 12-item survey (SF-12) in patients with back pain. Spine 2003; 28: 1739–1745. 10.1097/01.BRS.0000083169.58671.96 12897502

[23] Salyers MP, Bosworth HB, Swanson JW, Lamb-Pagone J, Osher FC. Reliability and validity of the SF-12 health survey among people with severe mental illness. Medical Care 2000 (38): 1141–1150. 11078054 10.1097/00005650-200011000-00008

[24] Larson CO. Use of the SF-12 instrument for measuring the health of homeless persons. Health Serv Res 2002; 37: 733–750. 10.1111/1475-6773.00046 12132603 PMC1434659

[25] Liang KY, Zeger SY. Longitudinal data analysis using generalized linear models. Biometrika 1986; 73: 13–22.

[26] Zeger SL, Liang KY. Longitudinal data analysis for discrete and continuous outcomes. Biometrics 1986; 42: 121–30. 3719049

[27] Montgomery AE, Szymkowiak D, Marcus J, Howard P, Culhane DP. Homelessness, Unsheltered Status, and Risk Factors for Mortality: Findings From the 100 000 Homes Campaign. Public Health Rep 2016; 131(6): 765–72. 10.1177/0033354916667501 28123222 PMC5230839

[28] To M., Palepu A., Matheson F. The effect of incarceration on housing stability among homeless and vulnerably housed individuals in three Canadian cities: A prospective cohort study. Can J Public Health 2016; 107(6): 550–555. 10.17269/CJPH.107.5607 PMC6972084 28252374

[29] Tsemberis S, Gulcur L, Nakae M. Housing First, consumer choice, and harm reduction for homeless individuals with a dual diagnosis. Am J Public Health 2004;94(4):651–56. . 15054020 10.2105/ajph.94.4.651 PMC1448313

[30] Goering P, Veldhuizen S, Watson A, Adair C, Kopp B, Latimer E et al National At Home/Chez Soi Final Report Calgary, AB: Mental Health Commission of Canada 2014 Retrieved from: http://www.mentalhealthcommission.ca (Accessed January 30, 2017).

[31] Novac S. On her own—young women and homelessness in Canada Ottawa: Ottawa: Status of Women Canada, 2002.

[32] Cheung AM, Hwang SW. Risk of death among homeless women: a cohort study and review of the literature. CMAJ 2014; 170(8): 1243–1247. 10.1503/cmaj.1031167 PMC385354 15078846

[33] Bassuk EL. The characteristics and needs of sheltered homeless and low-income housed mothers. JAMA 1996; 276(8): 640–646. 8773638

[34] Deschner A. Women and Homelessness in Canada, A brief review of the literature Montreal, QC: McGill University Centre for Research on Children and Families, 2015 Available at: https://www.mcgill.ca/socialdevelopment/files/socialdevelopment/women_in_homelessness_-_a_brief_report.pdf (Accessed June 30, 2017).

[35] Lake S. Kennedy MC. Health outcomes associated with illicit prescription opioid injection: a systematic review. J Addict Dis 2015; 35(2): 73–9. 10.1080/10550887.2015.1127712 26670724

[36] Zerger S. Housing: a fundamental component of drug policy. Int J Drug Policy 2012; 23(2): 91–93. 10.1016/j.drugpo.2011.12.001 22341721

[37] Kirst M, Zerger S, Misir V, Hwang S, Stergiopoulos V. The impact of a Housing First randomized controlled trial on substance use problems among homeless individuals with mental illness. Drug Alcohol Depend 2015; 146: 24–9. 10.1016/j.drugalcdep.2014.10.019 25465295

[38] Salem L, Crocker AG, Charette Y, Sero MC, Nicholls TL, Côté G. Supportive housing and forensic patient outcomes. Law Hum Behav 2015;39(3):311–20. 10.1037/lhb0000112 25365472

[39] Palepu A, Patterson ML, Moniruzzaman A, Frankish CJ, Somers J. Housing first improves residential stability in homeless adults with concurrent substance dependence and mental disorders. Am J Public Health 2013; 103 Suppl 2: e30–6. 10.2105/AJPH.2013.301628 PMC3969112 24148035

[40] Chavira D, Jason L. The Impact of Limited Housing Opportunities on Formerly Incarcerated People in the Context of Addiction Recovery. J Addict Behav Ther 2017; 1(1). 28713877 PMC5507072

[41] Gerlitz J, Joyce R, Gadermann AM, Hubley AM, Zhu SM, Stowe M, et al Observations from the Field: Interviewers and Retaining Homeless and Vulnerably Housed Participants in Longitudinal Health Research. J Soc Distress and the Homeless 2017; 26: 128–137.

