# Supplementary material for: The effect of forensic events on health status and housing stability among homeless and vulnerably housed individuals: A cohort study
Source: PLoS One. 2019 Feb 7;14(2):e0211704. doi: 10.1371/journal.pone.0211704 (PMC6366888; doi:10.1371/journal.pone.0211704)
Supplement: S1 File — (DOCX) [file pone.0211704.s001.docx]

**Supplementary Table A. Multivariate GEE Poisson regression model to estimate the independent effect of having an arrest on the number of residential moves**

| **Independent Variables** |  | **Adjusted RR for number of residential moves baseline to FU4 interview**  **(95% CI)** |
| --- | --- | --- |
| Forensic event* | Yes | **1.24 (1.19, 1.3)** |
| Age (by decade) |  | **0.95 (0.92, 0.97)** |
| Sex | Female | **0.93 (0.89, 0.98)** |
| Employment* | Yes | 1.05 (1.01, 1.10) |
| City: |  |  |
|  | Ottawa | 1.06 (1.01, 1.12) |
|  | Vancouver | **1.09 (1.03, 1.15)** |
|  | Toronto | Reference |
| Ethnicity | White | 1.05 (1.00, 1.10) |
| Homeless at interview time point* | Yes | **1.25 (1.20, 1.31)** |
| Interview time point* | | **0.83 (0.82, 0.85)** |
| Problematic Substance use* | Yes | **1.17 (1.12, 1.22)** |
| Ever had a mental health diagnosis | Yes | 1.01 (0.96, 1.05) |
| 3 or more chronic conditions | Yes | 0.97 (0.93, 1.02) |

* Time varying covariates

**Supplementary Table B. Multivariate GEE Poisson regression model to estimate the independent effect of having an incarceration event on the number of residential moves**

| **Independent Variables** |  | **Adjusted RR for number of residential moves baseline to FU4 interview**  **(95% CI)** |
| --- | --- | --- |
| Forensic event* | Yes | **1.30 (1.24, 1.36)** |
| Age (by decade) |  | **0.95 (0.92, 0.97)** |
| Sex | Female | **0.94 (0.89, 0.98)** |
| Employment* | Yes | 1.05 (1.00, 1.10) |
| City: |  |  |
|  | Ottawa | 1.06 (1.01, 1.12) |
|  | Vancouver | **1.08 (1.03, 1.14)** |
|  | Toronto | Reference |
| Ethnicity | White | 1.05 (1.00, 1.10) |
| Homeless at interview time point* | Yes | **1.26 (1.21, 1.32)** |
| Interview time point* | | **0.83 (0.82, 0.85)** |
| Problematic Substance use* | Yes | **1.16 (1.11, 1.22)** |
| Ever had a mental health diagnosis | Yes | 1.01 (0.97, 1.06) |
| 3 or more chronic conditions | Yes | 0.97 (0.93, 1.02) |

* Time varying covariates

**Supplementary Table C. Multivariate GEE Poisson regression model to estimate the independent effect of having forensic event in the interview prior to year assessing the number of residential moves (Lagged analysis)**

| **Independent Variables** |  | **Adjusted RR for number of residential moves baseline to FU4 interview**  **(95% CI)** |
| --- | --- | --- |
| Forensic event* | Yes | **1.35 (1.26, 1.44)** |
| Age (by decade) |  | **0.92 (0.89, 0.96)** |
| Sex | Female | **0.88 (0.82, 0.984** |
| Employment* | Yes | 1.00 (0.94, 1.07) |
| City: |  |  |
|  | Ottawa | 1.00 (0.92, 1.05) |
|  | Vancouver | 1.05 (0.97, 1.14) |
|  | Toronto | Reference |
| Ethnicity | White | 1.05 (0.98, 1.13) |
| Homeless at interview time point* | Yes | **1.43 (1.34, 1.53)** |
| Interview time point* | | 1.02 (1.00, 1.05) |
| Problematic Substance use* | Yes | **1.19 (1.12, 1.27)** |
| Ever had a mental health diagnosis | Yes | 1.00 (0.94, 1.07) |
| 3 or more chronic conditions | Yes | 0.98 (0.92, 1.08) |

* Time varying covariates
